# ‘Hell No!’—Exploring Scepticism in UK Health Research Since COVID‐19 Amongst Communities Who Have Been Labelled ‘Underserved’

**DOI:** 10.1111/1467-9566.70110

**Published:** 2025-11-07

**Authors:** Hannah Cowan, David Wyatt, Sven Smeets

**Affiliations:** ^1^ Department of Population Health Sciences King's College London London UK

## Abstract

Healthcare research globally has seen a renewed shift to increase diversity in research participation. People previously excluded from the production of biomedical knowledge, and often labelled ‘underserved’, are now a focus of attention. In this paper we discuss an in‐depth interview study in South London which aimed to better understand how the very public era of COVID‐19 research has affected people's trust, opinions and relationships with health research, focusing on hearing from those with intersectional experiences of inequality and injustice. We suggest that ‘underserved’, much like ‘diversity’, obscures historically rooted injustice with narratives of what Chandra Talpade Mohanty calls ‘benign variation’ and assumptions that health research has always worked in service to others. Rather, we draw on the work of Sara Ahmed to ensure we take participants' concerns, scepticisms or complaints about research seriously. Drawing on participants' narratives of health injustice, we document how participants embody critical dispositions, which demand more complex understandings of health research that incorporate doubts, nuance and multiple sources. Such accounts render into stark relief the underlying power relations in attempts to simplify research participation narratives. This study demonstrates research institutions need to engage in more complex dialogue with communities in order to be worthy of trust.

## 1 Introduction

Since the start of the COVID‐19 pandemic, healthcare research globally has seen a renewed shift to increase diversity in research participation, and ensure clinical research stands to benefit communities that have historically been excluded from the production of biomedical knowledge. In the UK, the National Institute for Health and Care Research (National Institute for Health Research 2020) frames this drive in terms of ‘reaching underserved communities’ to facilitate their involvement in research. This involvement includes acting as research participants, as well as drawing on their lived experience to advise on research and facilitate the research recruitment of others classed as ‘underserved’. The push to reach *all* citizens has been part of a national strategy to become a global powerhouse of clinical research for some time (Department of Health 2006; Department of Health and Social Care 2021, 2023). This work is grounded in a project to platformise the UK's National Health Service (NHS) and the patients it treats as a resource for researchers and the global bioscience industry, substantially contributing to national GDP through attracting private investment in UK research (Faulkner‐Gurstein and Wyatt 2021). This is most clearly described in the Department of Health (2006) report, *Best Research for Best Health,* which advocates embedding research into routine healthcare practice. Colloquially called the ‘health and wealth agenda’, this report framed research as not only benefiting the health of the population through medical advancements but also the wealth of the nation. Increasing diversity in research participation is an extension of this process, not only attempting to make research relevant to a wider pool of patients but incorporating diverse bodies that are seen as valuable, most notably those with racialised identities. Diverse people are here viewed as ‘assets’ or ‘human resource’ that bring economic value to powerful others (Ahmed 2007, 236), in this case increasing GDP through clinical research. This politico‐economic context of diversity does not dispute calls from critical social scientists that medical research needs to work beyond a white male model as norm (e.g., see Shim 2014). It does, however, help highlight why there are problems in how ‘improving diversity’ or ‘reaching underserved communities’ have been framed to date.

Work to bring ‘underserved communities’ into healthcare research is notably a long‐standing campaign in the UK (National Institute for Health Research 2020), which has included suggestions of making research materials accessible, ensuring there is representation of communities within research infrastructures, and improving patient and public involvement in research (Teodorowski et al. 2023). The NIHR INCLUDE project suggests ‘underserved’ can be defined as: ‘lower inclusion in research than one would expect from population estimates’ or ‘high healthcare burden not matched by the volume of research designed for the group’ (National Institute for Health Research 2020). While this broad definition may have been intended to incorporate different intersectional inequalities, it also enacts what Mohanty (2003, 193) describes as ‘benign variation’, which, like her critique of ‘diversity’, ‘bypasses power as well as history to suggest a harmonious empty pluralism.’ Similar to ‘diversity’, this definition of ‘underserved’ has the potential to be tokenistic because of how it obscures power relations, making differences in who gains from research appear random rather than historically situated (Ahmed 2012). Although there are some emerging and genuine attempts to incorporate historical injustices, including from within the NIHR network (e.g., see NIHR REPAG (Race Equality Public Action Group) et al. 2022), it is notable that the main institutional logics of ‘underserved’ do not consider or challenge the historical and political factors of how people become underserved in the first place. Without this understanding of how people become othered, the focus on immediate, practical ways to facilitate the inclusion of underserved individuals, often inadvertently ‘captures’ people with particular characteristics, categorising and assetising them in order to demonstrate a project's value (McKittrick 2020, 4). We suggest that the term ‘underserved’ also similarly obscures or misrepresents relations of power in how it portrays medical research as simply a philanthropic service, rather than something that makes profit, exploits, or simply something that requires time, collaboration, and intellectual and emotional action from participants themselves. Work, therefore, needs to focus more deliberatively on the power relations of research, looking not to diversity per se but rather distributions of power and knowledge; how people can share resources rather than be made to become resources themselves.

The COVID‐19 pandemic marked a significant shift in this landscape of ‘inclusion in research’, not least because it brought one of the most public and closely watched programmes of clinical research into people's homes globally. Crucially, this also means that what Stoler (2016) describes as ‘sediments’ of colonial logics in science rose to the surface and were made visible. Sometimes this occurred in overt ways like when medical researchers Mira and Locht suggested COVID‐19 vaccines first be trialled in Africa on French radio (BBC 2020). Equally, it occurred in more subtle ways, such as through the emergency lifting of data protections, where the origins of statistics as ‘state‐istics’ or data pertaining to government citizens belonging primarily to the state, became evident (Department of Health and Social Care 2022). The pandemic both worsened and made more visible intersectional inequalities, for the population in general, but young people in particular (Moore et al. 2021). People became more reflective of politics and how it plays out in daily lives and civil and political institutions. The reception of the vaccine has therefore not been unanimously positive. Survey data suggests the pandemic polarised the UK population, with those who experience structural injustice less likely to trust science (Skinner et al. 2020), and those, for example, who are neurodiverse, LGBTQ+, or people of colour are more likely to be ‘vaccine hesitant’ (Khorasani et al. 2023; Garg et al. 2021; Robertson et al. 2021).

This rupture has led to an emergence of literature, often aligned with community action, which looks to acknowledge and address inequalities through confronting relations of power in the research system itself. Community research organisations in South London where this study is based, such as Centric (2022), as well as academic teams such as Woodhead et al. (2021) and an NIHR REPAG (Race Equality Public Action Group) et al. (2022) have illustrated the ways in which histories of racialised abuse in research such as the Tuskegee trial, as well as present experiences of institutionalised racism, have led to vaccine hesitancy and a distrust of university‐led research. As Woodhead et al. (2021) explain, the pressure from the government for ‘Black and Minority Ethnics’ to take the vaccine could come across as suspicious, and as Goodman (2024) suggests, crossed boundaries into racialised policing through health. Key to this corpus of literature has been a critical turn from thinking about why or how people might trust research, to asking whether and how research institutions can be trustworthy (Best et al. 2021; Aitken et al. 2016), and whether they can take on the collective knowledge of communities (NIHR REPAG (Race Equality Public Action Group) et al. 2022). This shift puts the impetus on institutions to change the way they relate to communities, rather than attempt to continuously (and unsuccessfully) change people's views of research who have logical and rational reasons behind their distrust.

This paper builds on this literature, using interviews in South London to understand how the very public era of COVID‐19 has affected people's trust, opinions and relationships with health research. We focus not only on racialised injustice but communities who have intersectional experiences of inequalities and injustice. Whilst intersectionality has at times been blurred with the term diversity (Ahmed 2012), we take a critical approach to intersectionality, centring relations of power and how power is distributed (Piepzna Samarasinha 2018). We put particular focus on understanding people's doubts, scepticisms or complaints about research, including people's reasonings for being hesitant about the COVID‐19 vaccine, in order to better understand how institutions need to change. In thinking about scepticism, we draw firstly on work from Ahmed (2021) on ‘complaint’ as a feminist pedagogy which enables us to understand and critique power and how it operates. Ahmed examines complaints as distinctive communication forms and institutional processes, revealing broader structural issues. As she states, ‘[t]o hear someone as complaining is an effective way of dismissing someone’ (Ahmed 2021, 1) Complaints often get rendered invisible or dissipated by the very actors and mechanisms designed to investigate them. Our data and analysis do not engage with formal complaints per se but do engage with those whose opinions and experiences go against the status quo and are met with resistance from institutions and institutional actors. Here, we utilise Ahmed's work to take seriously the work involved in mobilising scepticism as complaints. We take scepticisms as forms of knowledge which attempts to make visible unsettling relations of power. Alongside this, we draw on the work of Goodman (2024) who suggests we should take vaccine rumours seriously. Rumours, she suggests, are not something simply fabricated from nothing but, as with complaints, tell us important truths about power relations in medical research and practice. In doing so, we aim to illicit decolonising narratives that emerge in popular discourse (White 2009), and infer what they mean for how research institutions need to change to gain a more widely held trust in healthcare research.

## 2 Methods

This research emerges from a small‐scale, exploratory project, funded through the NIHR South London Clinical Research Network's (CRN) ‘underserved communities fund’, which itself highlights the moment of introspection for health research that emerged from the COVID‐19 pandemic. We were awarded a small proportion of the available funds to better understand the thoughts and experiences of people living in South London who are not usually represented in clinical research, with a recruitment target of 10 interviews. We were committed to gaining in‐depth narratives from intersectional voices, particularly those who express scepticism of health research. We also wanted to assess how these narratives build on current understandings of participation and inclusion in local health research institutions.

We identified participants through three routes, and all participants were offered a £20 voucher of their choice to thank them for participating in the study. The first drew on young people and parents already known to HC through her South London based project, Utopia Now!, which explored how young people experienced the pandemic and envisage the future of health research and society. Through these contacts we purposefully sampled two young people and three parents who had previously expressed scepticism of health research. Of these interviews, one was conducted face‐to‐face with a personal chaperone who did not participate, one was conducted over the phone and included both Desta and her mum (Sandra) who joined in impromptu and later consented to participate, and two were completed over Microsoft Teams.

The second recruitment route used was Facebook advertising, which studies have claimed is useful for reaching ‘underserved’ communities (Whitaker et al. 2017). While it did allow us to reach a wider pool of potential participants, we also recognise that the pandemic exposed a barrier to digital access for some economically marginalised communities. Recruiting through previous community projects allowed us to reach people who have less digital access, but we recognise we did not entirely avoid issues of digital access and literacy (Pedersen and Kurz 2016). Using the existing Utopia Now! Facebook page and $30 of free advertising, the post was viewed by 4737 participants living in South London (according to Facebook metrics). However, we experienced a low conversion rate of advert views to interviews. To purposively sample our interviewees, we asked interested individuals to complete a short questionnaire, which emphasised that we would like to speak to people who had experience of inequality or injustice, and asked them to describe these experiences through an open text question. We also requested information such as age, vaccination status, and general attitude towards health research. Nineteen people fully responded to the survey with contact details and of these, one person was excluded on the basis of not living in South London. We began by approaching those who reported the most negative attitudes towards health research and that reported experiences of inequalities, but ended up contacting all potential participants due to a lack of response. We interviewed all six participants who were available to be interviewed from this sample over MS Teams.

Our third method of recruitment was focused on understanding current efforts to facilitate greater engagement with those labelled ‘underserved.’ We largely did this informally by attending regular meetings of Guy's and St Thomas' Research & Development Equality, Diversity and Inclusion Steering Group, and the South London CRN's Inclusivity Panel. We spoke to one professional involved in these groups through a formal interview over MS Teams to give us an overview of current concerns. Further details on recruitment routes can be found in Supporting Information S1.

We did not systematically collect the demographic data of participants using a form with pre‐defined categories as we did not want to replicate problematic categorisations of identity that were highlighted during ‘Patient and Public Involvement’ (PPI) sessions related to this study (See Supporting Information S1), and which act to ontologically ‘capture’ people as ‘other’ McKittrick (2020), 4). Indeed critiques of the way that studies which recruit through racialised categories because of the way they can make people feel targeted are also articulated by the participants of this study. Instead, we took on suggestions from PPI workshops (explained further in Supporting Information S1) that forms of inequality and marginalisation emerge from the environments people are living in, rather than simply from people's bodies and identities themselves. As we illustrate throughout this paper, these precarious marginalising environments, including the ‘discursive‐material’ can then get folded, or looped, into the body (Barad 2003) and embodied, both as affective registers (Blackman 2012, 23–24) and into material physiological health effects (Krieger 2016), but forms of inequality do not simply emerge from people's bodies and identities themselves. Rather than ‘capture’ specific groupings that have been collected and taxonomised through biomedical research (McKittrick 2020, 4), we have therefore reported experiential forms of inequality alongside our interview data which emerge from the environments in which people are situated and the stories in which people tell. We have summarised the positionality and forms of marginalisation that each participant speak to based on how they described it in the sign‐up form or interview itself in Table 1. All participants noted some experience of inequality or injustice, and include, but are not limited to: ableism, racism, insecure housing, neurodiversity, sexism, care roles, mental health difficulties and classism. Although authors of the paper have experiences of injustice relating to LGBTQ+ rights, these experiences are a notable omission from the data.

**TABLE 1:** Raising unheard voices study.

Participant (pseudonym)	Descriptive experiences of inequality and trust in research
Abby	Abby is a single mum who describes herself as white. She cares for a daughter who she describes as mixed race and has special educational needs. She works with multiple youth organisations in South London, and talks about her daughter's experiences of racism. She was more supportive of research, but described why people in her community were more sceptical.
Amara	Amara is a single mum who describes herself as Black and is aged between 35–44. One of her sons has autism, ADHD and a learning disability, and the other has ADHD. She is more sceptical of vaccinations and research.
Lyric	Lyric is a 17‐year‐old young woman who is doing a vocational training course. She describes her experience of autism and discrimination she has experienced around autism in the health service. She describes scepticism around health research and the COVID‐19 vaccine. She does not describe her ethnicity.
Declan	Declan describes himself as a Black man aged 39 who lives with his partner and two children. He decided not to get vaccinated but is ambivalent about health research and has taken part is some clinical studies in the past. He speaks broadly about institutional racism and healthcare inequalities.
Farzaneh	Farzaneh describes herself as a woman of colour who has grown up in South London. She is between 25 and 34 years old, and although describes trusting her local hospital, feels as though there was a shift in her trust in research during the pandemic.
Sade	Sade describes herself as a woman of colour aged 25–34, working in a lower paid role within a hospital setting. She describes institutional experiences of racism and the relationship between being a lower paid member of staff and ethnicity. She is more in favour of health research and vaccination but describes ambivalence about health research since the pandemic.
Shaun	Shaun describes himself as a white man who is a teacher and born and bred South Londoner. He describes himself as working class and currently lives with his wife in the same council estate he grew up in. He had his first COVID‐19 vaccination but describes becoming more sceptical as the pandemic went on.
Ethan	Ethan is a man between the ages of 25–34 who is vaccinated, but describes himself as becoming more sceptical of medical research since the pandemic. He does not explicitly describe his ethnicity but says he has experienced racism and talks about feeling more represented by public health adverts which are more ethnically diverse.
Obasi	Obasi describes himself as a Black man who is aged between 25–34 and is married with two children. He describes some experiences of racism. He decided not to get vaccinated and has ambivalent views about health research.
Desta	Desta is a young woman who lives with her mum and experiences multiple chronic health conditions. She describes having to go into emergency accommodation because of unfit housing conditions provided by a housing association. She also described the impact of the ‘cost of living’ crisis, and several negative experiences of hospital care. She does not describe her ethnicity. She is ambivalent about health research but is more sceptical of research around the COVID‐19 vaccine.
Sandra	Sandra is Desta's mum who joined in on Desta's interview which was held over the phone. She describes herself as a Black woman and describes difficult experiences and inequalities with housing authorities, hospitals and social services. She also has dyslexia. She describes scepticism of health research and the COVID‐19 vaccine.
Jiya	Jiya is a professional in patient and public involvement working within health research infrastructures of South London. We interviewed her to gain a better understanding of current thought and practice of research inclusion in South London.

*Note:* All descriptions are taken from interview conversation themselves or the sign up form where participants were asked to self‐describe their experiences of marginalisation.

We drew on existing literature to design the interview questions, exploring participants' trust in (public) institutions in general and health care in particular before moving into their knowledge and experience of health research. This was in order to understand how their views on health research are situated within their experiences of institutional inequality and injustice. We used the pandemic as a tool and lens to explore how research was communicated and public health initiatives were enacted, and asked particular questions about their views of efforts to increase representation. We see the very public research about COVID‐19 and the COVID‐19 vaccine as a time that heightened the senses of publics to health research, and a mutual point of reference which enabled multiple conflicting state‐sanctioned and counter‐narratives around health research to come into view. Our methodological approach to the study assumed that hesitancy around research and distrust in the COVID‐19 vaccine are reasonable and predictable responses which themselves express knowledge of the power relations of health research in general (Goodman 2024). We share understandings with Madorsky et al. (2021) who suggest ‘distrust’, a word that expresses a *rational* knowledge as the basis for deciding not to trust, is a more useful term to describe vaccine hesitancy. However, we also suggest that *intuition* or *suspicion* that form the basis of terms such as ‘mistrust’ and ‘hesitancy’ point to a more embodied and visceral way of sensing power which are equally valuable ways of coming to know the world (Blackman 2012). Interviews, therefore, encouraged participants to speak through the nuances, feelings and uncertainties in their narratives around health research, and COVID‐19 research in particular.

Interviews were audio recorded, professionally transcribed and anonymised (through participant numbers and then use of pseudonyms) and then independently analysed by all researchers. Our analysis process began with inductive coding completed by SS, making effort to stay close to the data for a descriptive account (Sandelowski 2000; Braun and Clarke 2006). These codes were developed into themes collectively over the course of three meetings which involved reflections on current literature around institutional distrust, and further analytical coding was then conducted by HC and DW. Initial findings were also discussed with members of our local NHS Trust's Research and Development Equality, Diversity and Inclusion Steering Committee. We focused our analytical gaze on institutional narratives and practices that make people question the trustworthiness of institutions and stop people from wanting to participate. Although there were two participants (Abby and Sade) who spoke more positively of health research, these accounts still presented nuanced points that speak to scepticism and wider issues of participant involvement. In the next section, we focus on the three most prominent themes.

Ethical approval was granted by King's College London Research Ethics Committee (MRA‐22/23–34516).

### 2.1 Embodied Scepticism: Understanding Trustworthiness From a Critical Perspective

Participants in this study largely reflected the literature which suggests negative historical and personal experiences of institutions in general can lead to a lack of trust in health research (Gainty et al. 2024; Woodhead et al. 2021; NIHR REPAG (Race Equality Public Action Group) et al. 2022; Centric 2022). These understandings were partially formed through intergenerational and diasporic knowledge of abuses in healthcare research towards Black people such as the Tuskegee trial, which is well documented in this literature:

as a Black man in England, I think there’s a lot of Black people who do not trust hospitals and health institutions for a good variety of reasons, whether it’s inequalities in health when it comes to Black and ethnic minorities. […] you have the Tuskegee trial in the States and how that went down. That has a domino effect for other Black people in other countries that therefore makes them not trust the health institutions in the countries they may be.

(Declan)

I don't think I'll be taking part in any medical research, like, this is fine. This is an interview. But like, say they needed, like my bone marrow or, you know, something, I don't feel confident, but also the historical links to, like, Black and ethnic minorities and medical research. That hasn't helped either. You know, like the syphilis trials […] That gives me a bit of scepticism.

(Amara)

Following Woodhead et al.’s (2021) work, participants suggested that the pandemic only exacerbated these tensions, as UK medical authorities chose to target ethnic minorities specifically in vaccine recruitment campaigns. Some participants felt like this targeting apportioned blame:

they were talking about the Black and ethnic minority not necessarily taking up the vaccination option as much as other demographics were. I wasn’t really too keen on that because […] it felt like sort of pressuring and targeting a community. […] I don’t think people should be called out for not taking it, […] especially when the narrative was being put out that if you weren’t vaccinated, you were putting people more at risk […] it’s like saying that these people are putting you at risk.

(Ethan)

Amara also critiqued this campaign, and later said of research in general: ‘don't just target one group because that's just suspicious to me’.

Interestingly, participants reflected the effects of wider institutional injustice from an intersectional lens, illustrating how multiple experiences of structural injustice can lead to people gaining a sense of critical awareness and scepticism of research. Farzaneh suggested they ‘just wouldn't want [their] data to be associated with a clinical institution’ for the purpose of research, emphasising the lack of trust they felt after the government's response to the pandemic. Furthermore, Desta and Sandra had a lack of trust in institutions to do right by them because of their poor experiences with disability support and serious problems with social housing during the pandemic. When asked about the vaccine they summarised that they did not want to take it because ‘they're killing us’ (Desta)—the ‘they’ standing in for multiple people and organisations in power and authority ‘not looking at the little people’ (Sandra).

For several participants, particularly women, conceptualisations of consent for research and medical interventions reflected narratives of sexual consent summarised by the idiom, ‘my body, my choice’. Talking of the vaccine, Lyric stated:

if you want to take it, that is your body, that’s your choice, you go and take it. And if you don’t want to take it, again it’s your body, your choice. You don’t have to take it.

(Lyric)

However, with the pressure and repeated messages experienced by participants, saying ‘no’ to vaccination, unlike norms for sexual consent, was not accepted as the end of the conversation.

This assertion for bodily autonomy extended to neurodivergent understandings of touch and sensory overload. Amara, who is a mother of an autistic son, suggested:

he's autistic and he's not just gonna freely give his arm anywhere. He doesn't even take oral medication.

(Amara)

Lyric also talked about her experiences of an ‘autistic meltdown’ and the lack of understanding for this need for bodily autonomy in healthcare in general.

While wider and historical systems may not always seem under the control of research institutions themselves, research‐intensive hospitals can ensure that people feel safe in their local hospital environments where they might get approached to take part in research. Desta had several bad experiences of attending hospital for Ehlers–Danlos syndrome, a condition where she experiences a high number of dislocations, leaving her ‘literally debating [whether to go to hospital] because I'm like, every time I go, something happens, or they call the social services [on mum].’ As she and her mum (Sandra) explained in conversation:

Desta This nurse even tried – erm, dislocated my shoulder trying to pull me out of a wheelchair that my ankle was dislocated and she told me to stand up on a dislocated ankle – […] they put me in a room that's for mental health people and not in the room to treat a dislocated ankle…

Sandra This nurse is inside – doctor is inside of the treatment room tugging my child out and I cannot get to her. So I had to run back and call for help. I ran into a room. There were people there and I said I need help, my daughter is being abused. Ohh we're in a meeting right now. No help, no help.

When Desta has then been told ‘oh you'd be a very good candidate for research’ she feels affronted: ‘After all I've done for you, helping you with research, now you want to actually physically test me?’ Her mother, even more sceptical, just responded:

Hell no! […] And who's to say that they won't do it? […] do they really need permission or will they do it whether or not? […] I don't trust them. If you can report me [to social services], who’s to say that you are not a law unto yourself.

(Sandra)

These experiences call into question the NIHR's strategy to make research a routine part of healthcare. This strategy only works if routine healthcare itself is a safe and trustworthy environment. Other participants cite experiences of being asked to take part in research that they deemed unethical, which also increased their scepticism of research as a whole:

So, I have autism. And I think last year or the year before I was nominated to go on this trial for autism. It was taking this pill that basically made you more socialisable or whatever it is. But I didn’t agree with that. It doesn’t really seem safe to put something in your body that will hopefully make you be able to talk to people. It doesn’t fit the picture, it didn’t make sense to me.

(Lyric)

This suggests research institutions not only need to look to institutional infrastructures and systems beyond and surrounding themselves but also within. Although historical injustice may seem out of reach for research institutions, both here and in national campaigns to target vaccinating ethnic minorities it is clear how the sediments of colonised knowledge build up in medical research infrastructures themselves (Stoler 2016). Supporting research studies that have not been discussed with communities could prove damaging to the research institution's overall reputation.

Participants present thoughtful accounts of why they may not trust particular institutions and research, which draw on a variety of community, experiential and other forms of knowledge from an intersectional lens. This not only illustrates how affective and ‘discursive‐material’ environments are folded and looped back into the body (Blackman 2012; Barad 2003) but that this embodiment results in alternative creative knowledge formations that ‘unfold into a different future’ (McKittrick 2020, 48–51). Participants’ accounts demonstrate scepticism as an embodied critical disposition, which brings about understanding of the possible intentions, evidence and underlying ideological motives, behind institutional discourses. Scepticism presented here is at once logical, based on evidence of past injustice and evaluation of current research and care, as well as something that looks to understand the felt affects of power (Blackman 2012). It is a reaction to power and knowledge formed through the looping of internalising and externalising experience that helps participants protect themselves against future exploitation. This supports the idea that there needs to be a shift from asking why particular members of the public do not trust research, to asking what makes research trustworthy, turning the gaze to institutions rather than on individuals who quite reasonably have some reservations about taking part in research (Best et al. 2021; Aitken et al. 2016). Most importantly, participant accounts suggest that respecting someone's decision to say ‘no’ to research or vaccinations could be even more important in the long run than convincing people to take part.

### 2.2 ‘Hesitancy’ as Making Time: Credible Knowledge Requires Space for Doubt

A key way in which participants worked with their scepticism was to think critically about the information that was presented and made available to them. This decision‐making process was particularly highlighted when it came to deciding whether to accept the COVID‐19 vaccine. Rather than just accepting statements about the importance and efficacy of the vaccine, participants made decisions about what sources were relevant and valid, what information they expected to receive and who they expected to receive the information from. In short, they reflected on what counts as credible knowledge.

…I did my own research. I was thinking, ‘shall I take it or shall I not take it?’ I did a week of really going into detail about the good and the bad of that vaccine. And I made my own decision. […] I went to the NHS website first. On the NHS website, it was saying blah, blah, blah, take it, take it, take it. […] Why are they not giving out as much information as they should be, but were just saying ‘go take it’? So, I did a bit of research about the bad sides of what affects your body, what it does to your body, things like that. So, for me, I didn’t end up taking it…

(Lyric)

What are the pros and cons? You know, I just really want to know, what are the cons of doing that [taking the vaccine]? You've just gotta weigh things up. That's all, it's about weighing everything up […] there's pressure, just putting the pressure on, like, get the vaccine, and these groups need to get it now, like, no. I don't need to get it urgently […] everything just felt like it was done really fast.

(Amara)

Whilst there are well documented accounts of misinformation in regards to the COVID‐19 vaccine that these participants may or may not have looked into (Gabarron et al. 2021), these accounts point to truths about power: that government information on vaccines is also scripted to influence publics in particular ways. The lack of nuance in official information led participants to carve out time to research the COVID‐19 vaccine beyond government messaging, to think critically and actively consider whether to be vaccinated.

Shaun also highlights the importance of experiential knowledge in decision‐making, and pressure to be vaccinated even when someone has had a bad reaction to the vaccine.

I’ve sadly become a little bit more sceptical of the vaccine programme and that’s based on experiences with my mum. As I said earlier, she was a nurse. And she reacted very badly to the first – she had her first jab and she reacted very badly. Her blood pressure dropped and she collapsed in the middle of A&E. […] it took a whole fight for her work to accept that she was not going to have the second jab.

(Shaun)

These reflections were also situated in a context of what participants experienced as ‘scaremongering’ to promote vaccination:

I think there was a lot of panic during the first phase of COVID. And I think there was a lot of scaremongering as well. And I think that’s what contributed particularly as well to the whole unvaccinated‐vaccinated divide in the community. So, like for me, I am not vaccinated. And I definitely had heated discussions with people about vaccinating. Yes, I think I can see why there’s mistrust especially for people that are unvaccinated. […] The vaccinations were fast‐paced, and not really tested.

(Declan)

Narratives, such as the one expressed by Declan, were not limited to those who were unvaccinated. Numerous participants stressed the use of pressure and rhetoric to push people to get vaccinated quickly, where ‘literally, you got seven texts a day on your phone’ (Desta) and:

you’ve got a narrative that’s put out there and it’s almost like you can’t question certain points, so that comes out – or even with vaccinations. I’m vaccinated, so, I’m not like a conspiracy theorist or an anti‐vax or anything like that. But like, that, so for me was because of the fear‐mongering that I feel like was going into things […] there was a really intense period of ‘you have to have it, you have to have it, you have to have it,’…

(Ethan)

This emphasis on the speed of decision‐making was also stressed more broadly in the context of medical research participation:

They’re told about research within like 5 minutes. So, they might not feel like they’ve had enough time to kind of think about what it is. So, it’s just those kind of things where I think people feel like, ‘If we had a little bit more time and a little bit more information, then actually we would be able to make a choice.’

(Jiya [community engagement professional])

These accounts of COVID‐19 and research participation highlight the importance of giving people time and sufficient information to decide about vaccination and research participation. What is noteworthy in the context of the vaccine specifically, however, is how vaccination, often something that is a choice (even if a contentious choice), was presented and experienced as a directive rather than a decision. The lack of nuance in public dialogue, particularly about the potential negative impacts of the vaccine, was also highlighted by many participants. These accounts stressed the need for an acknowledgement of the known unknowns, the risks and the benefits. A specific point of concern related to the disconnect between public discourse on medical care, namely, the image of the individual as unique and requiring personalised medical treatment, and a vaccine that was presented as a one‐size‐fits‐all solution:

everybody's body is different. Everyone can react differently to different vaccinations. We're not all the same. We're not clones. We've all got different DNA. […] And we react differently to medication. We're all being treated in the same pot, treated the same […] we don't know what the long‐term effects are going to be on our general health and our wellbeing, and especially when you care for young people with, like, needs, how it's going to affect them like neurologically or, you know, their genetics. And just long‐term.

(Amara)

I said, well, why are you giving the vaccine to everybody with different needs, different allergies […] I am scared because I've had pneumonia, I had whooping cough. […] And you want to stick me with this injection and you don't have my medical history in front of you, and you want me to take this vaccine? I'm scared. ‘Oh there's nothing to be scared about, it's safe and wawawa.’ […] I got the whole works. So why can't I take my daughter's medication? You're telling me no, but you're giving us all the same vaccinations. […] One shoe does not fit all.

(Sandra)

Participants' accounts highlight how credible knowledge or credible claims require acknowledgement of uncertainty and doubts. In attempting to simplify information and encourage people to take the vaccine, government messaging left little room to acknowledge uncertainty, or even outline the potential risks or side effects, highlighted by Amara, that are usually given with every medication. Taken together, government messaging on vaccines has left some of our participants more sceptical of research. When asked directly about whether they trust health research, Shaun remarked:

The pandemic has changed it. I would have once said, yes. Now I’m sceptical. I would have once said ‘why would a health body have a nefarious reason for anything? Why would they lie?’ Because of the pandemic now, the whole mismanagement of it and everything else, I’m, yes, I’m not sure, to be honest. I don’t think I do.

(Shaun)

Crucially this messaging highlights an institutional obliviousness to the embodied scepticism presented in the previous section, the critical disposition of those who have historically and experientially known injustice through health research. These accounts demonstrate that decision‐making is contextual—and those who have developed an embodied scepticism towards institutional information require more depth, nuance and transparency in the information they receive. Whereas ‘vaccine hesitant’ is often used institutionally to describe many of our participants, what we see here is individuals who took time to research and reflect, who drew on a variety of sources to make decisions and who expected transparency (operationalised as balance and honesty) about the risks of the COVID‐19 vaccine. These individuals made active, thoughtful decisions based on the information they deemed credible, and the information made available (and that they viewed as conspicuously absent) in formal channels. Their ‘hesitancy’ is a reasonable temporal adjustment that refuses institutional time pressures to simply say ‘yes’, and is also in part a product of the lack of trustworthy practices in presenting and promoting the vaccine to them.

### 2.3 Considering Institutional Allegiances: Building Trustworthy Institutions

During interviews, participants suggested numerous ways institutions could begin to rebuild trust, but this was invariably seen as a slow difficult process. It became apparent in interviews that research institutions' allegiances with communities may not necessarily align neatly with other institutional allegiances, namely to government‐level policies. Rather, participants highlighted tensions between commitments of local NHS research hospitals to local communities and national or global research agendas that were followed in the pandemic:

before COVID, I think generally institutions like hospitals and universities that do medical research, I think they were looked upon in a positive light. […] But then, I think, when the COVID vaccine came about and there was a race then to get them to a working level, and then we started seeing people having health issues. I think that’s where the trust for the institutions started to dwindle a little bit.

(Declan)

the problem is you’re under a massive umbrella and it’s not a good umbrella to be under because you’re under the World Health Organisation, you’re under the NHS management, […] that damage[s] your reputation by proxy. […] [Y]ou are an injured organisation which sadly has been conflated with government and bodies like the World Health Organisation […] And now, if I think about any medical research, I’m going to think, ‘What’s your angle, who’s making money out of it, how corrupt is it?’ And that’s not the way it should be.

(Shaun)

Shaun articulates well the feeling other participants also had about the level of control overarching institutions had over their local GPs (Amara) and healthcare staff (Lyric and Declan), with a general feeling that local hospitals had to toe the line. Concerns about the power and influence the government has over local research institutions extended to concerns over the profit motives and involvement from corporate partners. Similar to Shaun above, other participants also felt like allegiances to private industry in the pandemic had made them more sceptical of healthcare research in general:

there was a distrust, definitely, when it comes to the vaccine and stocks and shares involved in, like, AstraZeneca and all those providing the vaccine, just noticing things about stocks and shares, so it's all very political also, like, very like personal, whereas people just all about money grabbing. So human wellbeing, profit before people, basically, that's how I felt.

(Amara)

You start seeing funding being organised by techs and, when you start looking into who knows who, in the sense of beneficiaries, it will always be benefitting their friends who own these companies and stuff – then you start, yes, it just makes you feel like very negative about the situation.

(Ethan)

However, as Ethan continues, there is some hope. They, along with other participants, suggest this hope lies in the distinction between local research institutions and larger for‐profit companies:

There’s more trust [in local institutions] in the sense of, feels like there’s more separation from the big corporate – let’s say like a big pharmaceutical company that has, feels like they have unlimited power and influence. Whereas I feel like people working in medical research and like the university, on the ground, local level, like even though it’s still a big institution and maybe mentally it feels like you still have people with integrity that are in touch with regular people and trying to still have an impact, a positive impact rather than lining their pockets.

(Ethan)

I definitely trust the research that’s happening at the hospital as opposed to a multi‐national organisation.

(Farzaneh)

let me start with academic institutions, […] I wouldn’t think they would be any ulterior motive for any form of research. […] There would not be any form of money‐making mindset behind the research. For hospitals, it can be, it can be both ways. I know sometimes hospitals work with pharmacies in terms of the supply of drugs and all that. […] So, I would say, my trust can’t be absolute because some can be money motivated.

(Sade)

While it is worth noting that these participants might not be aware of the growing number of partnerships between research institutions and private industry (Faulkner‐Gurstein and Wyatt 2021), it is interesting to note the hope and belief that local researchers can be more trusted *because* they act independently of private finance and national policy. Some participants, did however, suggest it was going to take local institutions some time to repair relationships with communities after the pandemic:

You can’t force it and you can’t say, ‘right, let’s do this campaign’ […] you can’t force people to trust you on your time, sorry. […] The only thing that you can do is a very long‐term project which involves remaining scandal‐free, transparent, professional, and competent. And by that, you’ll gain your own reputation. But this isn’t a quick fix thing you’ve got on your hands here […] any quick fix you do, people are going to be sceptical of anyway and then you’re back at square one.

(Shaun)

it's a bit like a foundation, you can't put a house on top of a hole. It needs to be built up, so you need to have the diversity first in the hierarchy. And then you slowly build your case […] Like asking people, I have this research going, if you're not interested, what would you be interested in researching?

(Abby)

Building these allegiances with local communities and listening to what people have to say reflects the current literature on building trustworthy research institutions (Best et al. 2021; Aitken et al. 2016). However, here we see that this is also a slow process, one that takes time and is evidenced through action. It is also apparent that forming allegiances with communities—listening and taking their suggestions into action—may mean that research institutions need to withdraw allegiances elsewhere, or else remake these relations with the support of communities. Ultimately, building trustworthy research systems requires a long‐term commitment and realignment of research to work collaboratively with, and become a trusted part of, local communities:

…obviously we also say like, ‘We’re going out in the community.’ […] maybe the focus now needs to kind of be more part of – we are also part of that community, and we want to maybe kind of work that way.

(Jiya [community engagement professional])

## 3 Discussion

Calls to ‘increase diversity in research’, especially for those who are ‘underserved’, are increasingly prominent within medical research infrastructures globally. This paper looked at the very public COVID‐19 research as a moment where both state‐endorsed campaigns, and counter‐narratives around involvement in research gained greater traction. In particular, we looked at emergent understandings of medical research and the COVID‐19 vaccine itself from people with intersectional experiences of inequalities, marginalisation and social injustice in South London. We see complaint as a crucial narrative form and practice for critiquing unfair or unequal relations of power (Ahmed 2021). Complaint, we suggest, can be a formal process, as well as one that can emerge in more implicit counter‐discourse or even what Goodman (2024) might call rumour. In any case, these counter‐narratives can play an important decolonising role (White 2009), by eliciting knowledge on how power is utilised. We therefore looked to narratives which expressed a lack of trust in COVID‐19 research, and to what truths these narratives might tell of more general power relations in medical research infrastructure.

Our research supports previous findings that historically embedded experiences of institutional racism can inform people's assessments of the trustworthiness of research institutions (Gainty et al. 2024; Woodhead et al. 2021; NIHR REPAG (Race Equality Public Action Group) et al. 2022; Centric 2022). However, participants in this study suggest the decision to trust institutions can not only be informed through a diasporic knowledge of racialised injustice, but also across intersectional experiences of injustices around gender, neurodiversity, disability and class. This paper therefore extends current research to look at shared experiences of injustice and how these experiences can contribute to a critical disposition towards research and institutionalised power in general. This is, of course, not a totalising relationship between trust in research, trust in the COVID‐19 vaccine, and experiences of marginalisation but one we suggest should be taken into greater consideration by research insitutions who want to engage more with those who have been marginalised. For participants in this study, this critical disposition is developed through intensive research, and shared experiential knowledge of the ways in which public health institutions and medical organisations, past and present, have not worked in the interest of those they label ‘underserved’. Rather than engaging with these histories, participants felt state infrastructures contributed to making a divide, and problematised those who were ‘hesitant’. However, we suggest ‘vaccine hesitancy’ reflects a resistance to the speedy temporal pressure of COVID‐19 research and vaccine uptake (Wyatt et al. 2021) and is a crucial act of making time to understand and evaluate multiple sources of knowledge, rather than solely following the orders of those in power.

Significantly, the objection of ‘hell no!’ and the careful boundary makings of the body illustrate how this critical disposition can also be built from affective knowledge, and embodied intergenerational and diasporic memories, of being mistreated by health professionals and researchers (Blackman 2012). Participants' accounts of bodies being overwhelmed, pushed and moved in non‐consensual spaces illustrate the potential bodily effects of institutional power. This power may not be ‘seen’ or acknowledged by everyone in the room, but represents knowledge formations gained from the relational embodiment of oppression (McKittrick 2020, 48–51). For our participants, this affective knowledge of power enables a pause, a shift in temporal approach to knowledge which acts as a catalyst for participants to think critically through the information they have at hand. Embodying inequalities here then, is not only about embodying the affective, discursive and material impacts of discrimination and injustice (Blackman 2012; Krieger 2016; Barad 2003), but is also about forming creative critical analysis that ‘unfolds into a different future’ (McKittrick 2020, 48–51), including a critical disposition towards power and particularly institutions that claim to serve.

As Ahmed (2021) suggests, this way of knowing the world is not an easy one to uphold, which further illustrates the power of research institutions. Although not all of the participants in this study raise formal complaints per se, they are articulating their understandings and opinions that go against institutional accounts and expectations, and in some cases asserting their bodily autonomy in doing so. Ahmed's work sensitises us to the labour involved in speaking directly to power, and participants' accounts demonstrate the impact and aftermath of these activities. Sandra, for example, was faced with being reported to social services after complaining about the care being given to her daughter, Lyric described the repeated pressure to take the vaccine after they refused, and Ethan documents the way Black communities were portrayed by authorities as guilty of spreading the virus. For these participants, saying no to the COVID‐19 vaccine was not easy or rash. Going against the institutionally recommended or sanctioned norm (i.e., vaccination or research participation) takes substantial work, and still there is fear that saying ‘no’ to research may not be respected while being treated in the NHS. Here, participants highlight the incongruence between the underlying notion of being ‘served’ behind emerging policies trying to increase involvement of the ‘underserved’ and how such policies are enacted and experienced in practice. So long as research institutions assume themselves to be benign actors, working purely in service of patient and public benefit, then it is unlikely that these policies to increase representation alone will address these kinds of underlying issues.

We suggest future work needs to shift the critical gaze, focusing not on ‘the underserved’ but those who claim to ‘serve’. Whereas some may question the validity of the sources some used to counterbalance official COVID‐19 vaccination messaging, with a history littered with untrustworthy practices, the trustworthiness of institutional sources and actors are also deserving of critique, and require consistent ongoing work. This is particularly so in light of nepotistic relations with individuals and private industry that were rendered visible to participants in the pandemic (McKee 2023; Iacobucci 2024). Rather than problematising those whose thoughts and actions render the lack of trust visible, we foreground the need to create an environment that is, and institutions that are, worthy of trust. One way of doing this might be to facilitate space for ‘healthy scepticism’ (see, Gainty et al. 2024). We build on this by thinking with Ahmed (2021, 4), who suggests listening with a ‘feminist ear’, which ‘is to hear what is not heard, how we are not heard.’ For example, listening with a feminist ear allows us to draw into stark contrast what Ahmed and participants in this study criticise as empty language and imagery of ‘diversity’ to call on particular categories of people to join research uncritically, and the active and often exhausting, invisible work and emotional labour of navigating institutions that have, and continue to, exercise harm. Rather, listening with a feminist ear might enable institutions to consider the multiple decisions, alignments and allegiances they make, together with efforts to build more trustworthy relations with communities. Although medical research is being positioned as something which brings both ‘health *and* wealth to the nation’, what is ‘not heard’ is whether it is always possible for the two to be mutually aligned. Working with communities would enable institutions to consider their holistic research portfolios, questioning: who stands to benefit? Who is research ultimately there to serve?

Rather than acknowledge this ‘healthy’ scepticism, participants perceived public health messaging in the pandemic as giving a strong and simple message, with doubts about who the vaccination stood to benefit surveilled, critical dispositions suppressed. Through the lens of Ahmed's (2021) work on institutional complaint, these accounts suggest that health research institutions made those doing the complaining the problem in an attempt to wash away or dissipate complaint, which only led to further depletions in the trustworthiness of these institutions. Conversely, central to the notion of building trust for participants were ideas of transparency—specifically articulated as communicating nuance and doubts over certainty. Indeed, certainty was seen as unrealistic and there to hide alternate truths and realities. As Cohn et al. (2013) argue, it has become a trope of biomedical research to sift the complexity out—to give simple unifying answers. Even when complexity is articulated, it is made stable, still in time, in order to navigate it rather than account for dynamic moving parts which are continually remade in relation to one another. Guidelines around public participation in research often advocate the use of ‘plain English’, when communicating research with publics (Health Research Authority 2024). However, this has perhaps been conflated with simplifying complexity out, rather than explaining complexity, nuance and doubts in straightforward language. Good research and good knowledge rarely deals with certainties or facts that can be forever stabilised in time. Research, by its very nature, means knowledge is constantly evolving, and so it is difficult to believe in a vaccine without potential for side effects or future knowledge coming to change what we knew in the past. Through ignoring, or indeed, surveilling, dismissing and attempting to suppress critical dispositions, research institutions have perhaps misunderstood the kinds of scepticism that many people who experience social injustice embody to avoid future harm to themselves.

Relationships between institutions, researchers and local people require slow on‐going work, and trust is earned through trustworthy practices; trust cannot be taught, assumed or demanded. Research institutions need to articulate and be transparent about the complexity of research, work more closely with communities and reflect on what messages the practices of research infrastructures as a whole tell those who have been marginalised.

## Author Contributions

**Hannah Cowan:** writing – original draft (equal), writing – review and editing (equal), formal analysis (equal, lead), investigation (supporting), methodology (equal) conceptualisation (equal), supervision (equal), funding acquisition (equal). **David Wyatt:** writing – original draft (equal), writing – review and editing (equal), formal analysis (equal, lead), investigation (supporting), methodology (equal), conceptualisation (equal), supervision (equal), funding acquisition (equal). **Sven Smeets:** investigation (lead), data curation (equal), formal analysis (supporting), methodology (supporting), project administration (equal).

## Funding

This research was funded by the NIHR South London Clinical Research Network's ‘underserved communities fund’ 2022–2023.

## Ethics Statement

King's College London Research Ethics Committee (MRA‐22/23‐34516).

## Consent

All participants consented to participation in this research.

## Conflicts of Interest

This research is funded through the NIHR to explore research participation of ‘underserved communities’. This presents some conflict of interest but this was discussed and reflected on in the research paper itself.

## Supporting information


Supporting Information S1

## Data Availability

Research data are not shared.
