# Supplementary material for: ‘Hell No!’—Exploring Scepticism in UK Health Research Since COVID‐19 Amongst Communities Who Have Been Labelled ‘Underserved’
Source: Sociol Health Illn. 2025 Nov 7;47(8):e70110. doi: 10.1111/1467-9566.70110 (PMC12595165; doi:10.1111/1467-9566.70110)
Supplement: Supplementary file 1 — Supporting Information S1 [file SHIL-47-0-s001.docx]

**“Hell no!” – exploring scepticism in UK health research since COVID-19 amongst communities who have been labelled “underserved”**

**Appendix 1**

**Our rationale for understanding marginalisation differently:**

The decision not to collect demographic data using traditional questionnaire methods was informed by wider participatory work carried out by [AUTHOR 1] and [AUTHOR 2] in South London. For both [AUTHOR 1] and [AUTHOR 2] this included interacting with [ANONYMISED HOSPITAL] wider Patient, Public, and Involvement groups around research. [AUTHOR 1] also engaged more broadly with communities in South London through co-leading two related participatory research projects called [ANONYMISED PROJECT 1], and [ANONYMISED PROJECT 2]. [ANONYMISED PROJECT 1] is a creative participatory project which looks to compare the futures currently being envisioned and enacted within medical research, with the hopes and fears of young people with intersectional experiences of marginalisation in South London. [ANONYMISED PROJECT 2] emerged during the pandemic, as community research partners wanted us to focus on how young people were being impacted by lockdowns. These projects not only helped the “Raising Unheard Voices” project with recruitment, but helped us conceptually shape the project through three years of prior engagement with community stakeholders and people living in South London on issues of research.

One of the key learnings from this participatory work that informed this project included a feeling that Black and global majority people of colour in South London had been targeted both by police and public health professionals during the pandemic. This included examples described in interviews in [ANONYMISED PROJECT 2] of police moving on groups of young Black people who were congregating above the legal number, but leaving adjacent groups of young white people who were over the lockdown cap alone. It also includes accounts in PPI groups of people worrying about Black communities being targeted for experimentation with the COVID-19 vaccine, and describing undue pressure on Black people to take the COVID-19 vaccine. Some of this narrative emerges in the study data itself, where we were told researchers should not just target specific groups of people as it feels suspicious, and puts particular pressure and blame on them to participate. This meant we did not want to specifically target racialised or any other groups to talk about marginalisation or mistrust in research, and rather let people self-describe why they felt an injustice and how this related to their views and thoughts about health research.

As part of [ANONYMISED PROJECT 1], [AUTHOR 1] also held a workshop at the [ANONYMISED NAME AND LOCATION OF THE EVENT]. This workshop brought together data scientists and students, including those from the university’s LGBTQ+ society. The group included people with intersectional experiences of marginalisation, including racism, genderphobia, ableism, and homophobia. The workshop invited the group to explore their identities; how they feel seen by others, how they would like to be seen, and how they feel represented in standardised demographic surveys. We did this through a series of creative participatory activities including zine making about ourselves, and graffitiing over current demographic surveys with what it provoked us to say, rather than feel the need to fill the boxes themselves. During the closing discussion, participants put forward different ways to think about how we should talk about identity and marginalisation in research. One suggestion that emerged was to allow people to self-describe their identity, rather than have fixed categories and options that are chosen for them. Others suggested we look to measuring marginalising environments – because the inequalities emerge from unequal environments rather than from having a particular identity.

In this study, we therefore decided to experiment with ways to work with and talk about identity and marginalisation which didn’t simply replicate categories and methods of ‘box ticking’ that those we worked with found problematic. We didn’t want to replicate forms of data collection that reproduced marginalisation, and particularly didn’t want to extend the feeling of Black and global majority people of colour people in South London being targeted by public health responses to COVID-19. We decided, instead, to take on McKittrick’s (2020) challenge of being more creative with research methods, to adapt our research practice, and draw on learnings from the workshop. We asked more open-ended questions that allowed people to describe their own experiences of inequality and injustice, both in an open-text form and in the interview itself. Participants described these experiences in their own words, identifying what they saw as experiences of inequality and injustice. Foregrounding participants’ experiences and classifications, we put control of the narrative in their hands. This also proved fruitful in providing space for participants to identify experiences of inequality and injustice that may have been missed in standardised categories (for example, participants Desta and Sandra described housing-related marginalisation). Our approach allowed people to self-describe their identity and to think about intersectional marginality as emerging from environments, rather than feel targeted themselves.

**Recruitment:**

The Facebook advertisement (which recruited 6 of our participants) had rules on whether any protected characteristics could be used in the text of the advert. We therefore had to keep this advertisement more general. Those who contacted us were then sent to information sheet and survey above so that they could consider taking part. For the text used, see *Figure 1* below.

**Raising Unheard Voices on health research in South London**

**Do you trust researchers to find new healthcare treatments?**

**How has the COVID-19 vaccination research, and the government's response to COVID-19, affected people's trust in universities and healthcare?**

If you have something to say, then we want to hear from you.

We are holding interviews with adults (16+) living in South London which will last for about 1 hour, and can take place in person or online.

You will be compensated with a £20 voucher for your time.

If you would like to take part, please get in touch with [researcher’s email address].

*Figure 1: Facebook advertisement text*

People were then led onto a sign-up form which more explicitly started by stating we want to hear from those who feel as though they experience marginalisation. We asked them to consider and respond to two questions, presented in *Figure 2*.


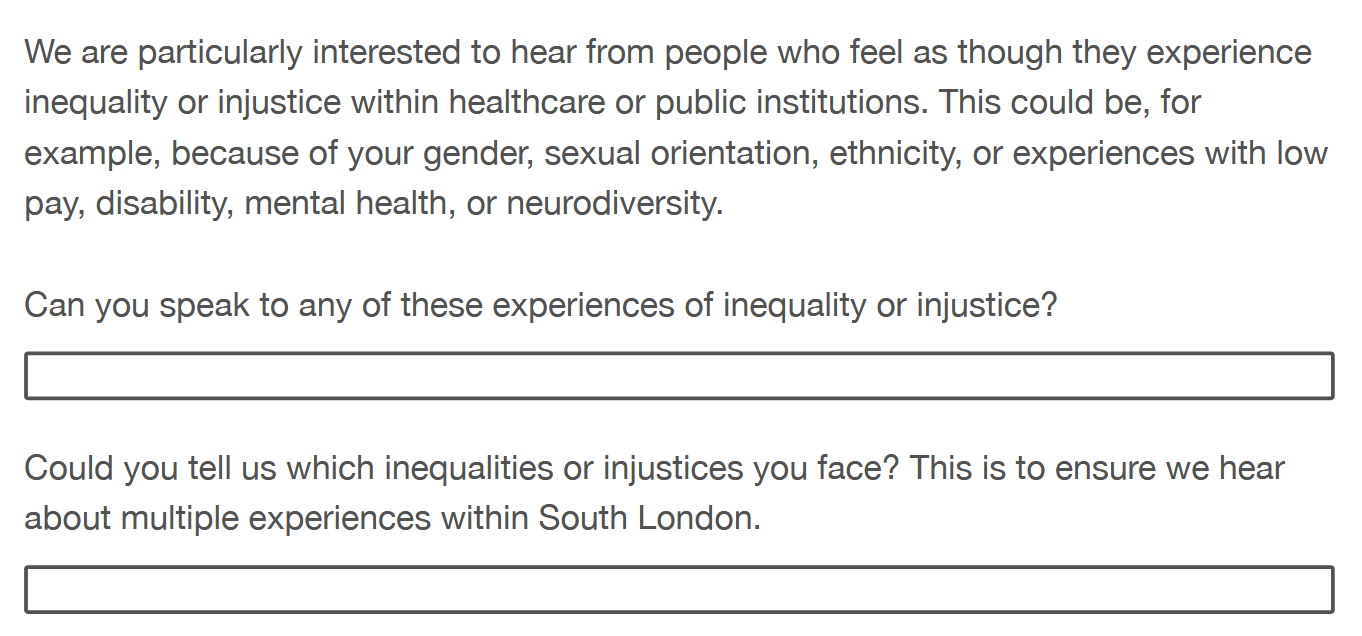


*Figure 2: Sign-up form text on experiences of inequality and injustice.*

Participants were then sent an information sheet which also made clear we want to hear from those who experience marginalisation. This text is included in *Figure 3.*


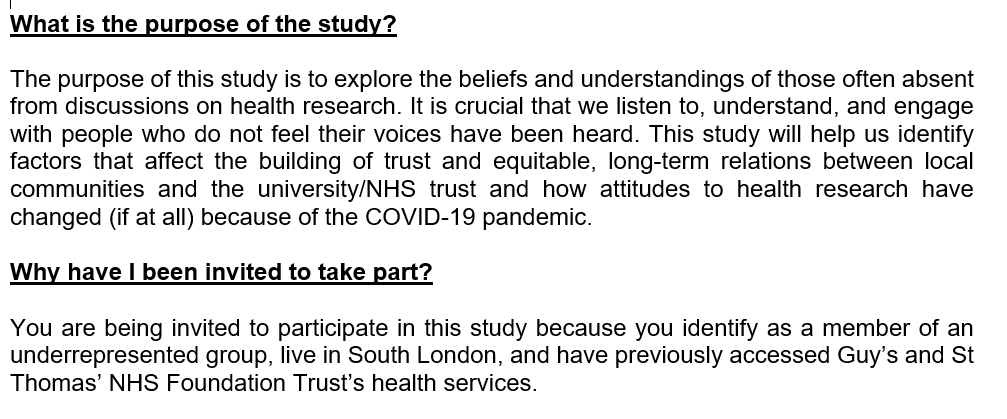


*Figure 3: Participant information sheet description of the study*

**Recruitment Flowchart:** In *Figure 4* below, we present a flow chart that documents the **
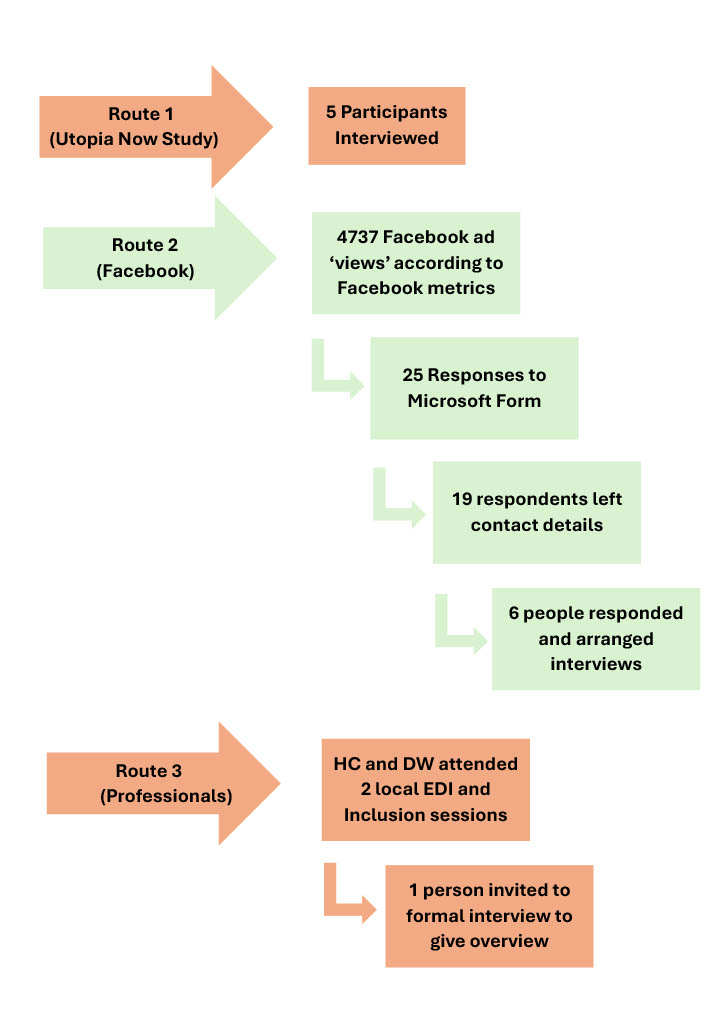
**recruitment process and the drop out at each stage.

*Figure 4: Flowchart of participant recruitment process*

**References:**

McKittrick, K. (2020) Dear Science and other stories. Duke University Press.
